# Supplementary material for: OneFlowTraX: a user-friendly software for super-resolution analysis of single-molecule dynamics and nanoscale organization
Source: Front Plant Sci. 2024 Apr 19;15:1358935. doi: 10.3389/fpls.2024.1358935 (PMC11066300; doi:10.3389/fpls.2024.1358935)
Supplement: Supplementary file 1 [file Image_1.pdf]

frontiers

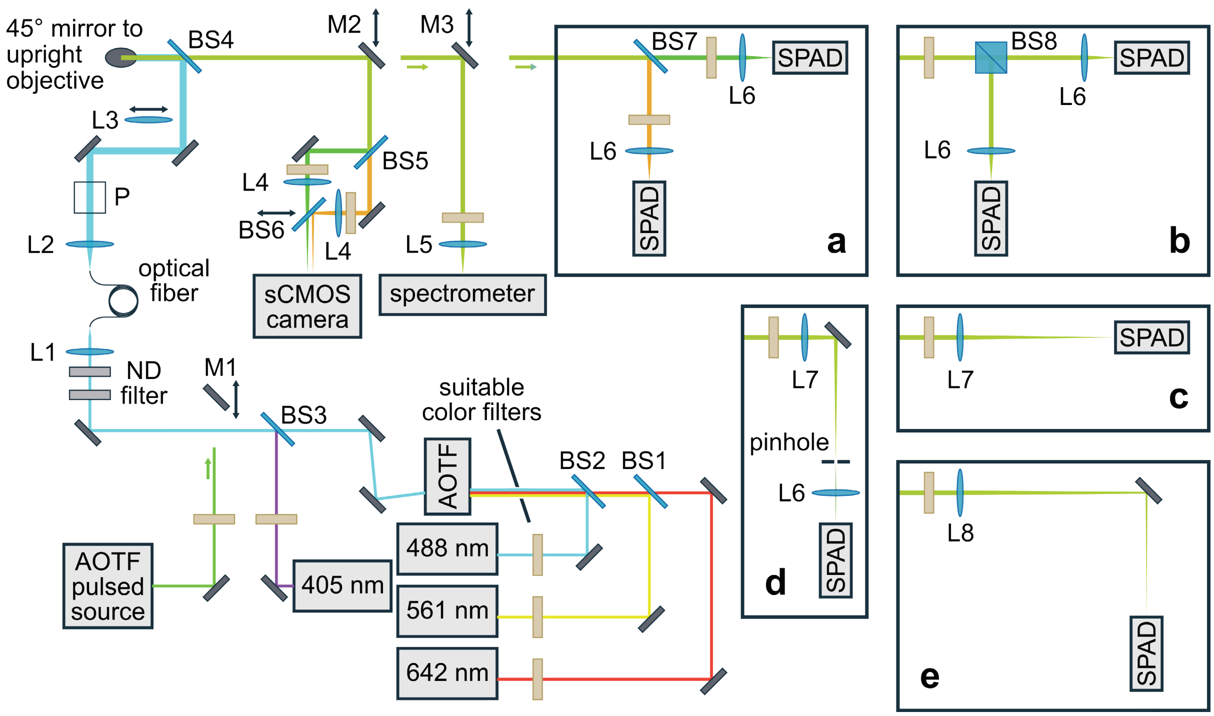

Supplemental Figure 1. Compact schematic overview of the research microscope.
For a detailed description and references to numbered items like lenses (L), beam splitters (BS) and mirrors (M) see text in Supplementary Materials and Methods, also for the different confocal configurations depicted in insets (a-e). Abbreviations: Acousto-optical tuneable filter (AOTF), neutral density filter (ND filter), sCMOS (scientific complementary metal-oxide-semiconductor) camera, single-photon avalanche diode (SPAD). The box P in the beam path denotes the position for additional elements, such as polarization optics. Adapted from (zur Oven-Krockhaus, 2021)

References

zur Oven-Krockhaus, S. (2021). A modular, comprehensive microscopy platform for modern live cell imaging. Thesis. https://doi.org/10.15496/publikation-62250.
