# Supplementary material for: OneFlowTraX: a user-friendly software for super-resolution analysis of single-molecule dynamics and nanoscale organization
Source: Front Plant Sci. 2024 Apr 19;15:1358935. doi: 10.3389/fpls.2024.1358935 (PMC11066300; doi:10.3389/fpls.2024.1358935)
Supplement: Supplementary file 2 [file Presentation_1.pdf]

Supplementary Materials and Methods

1 Microscopy

All sptPALM measurements were performed on a custom-built microscope platform, designed for interdisciplinary research approaches, using different spectromicroscopy techniques. The following description will therefore also include techniques beyond sptPALM. A complete description of this microscope and its application in various research projects can be found in (zur Oven-Krockhaus, 2021). References to specific modules and components refer to Supplementary Figure 1.

1.1 Excitation module

Four different continuous wave (CW) lasers are implemented in the main excitation path: 405 nm (iBeam smart 405-S-LP, 100mW, Toptica), 488 nm (Oxxius Laserboxx Diode laser, 100 mW, OXX-488-100, Laser 2000), 561 nm (Vortran Stradus DPSS laser, 50 mW, VOR-561-050) and 642 nm (Oxxius Laserboxx Diode laser, 130 mW, OXX-642-130). They cover the most common range of available and utilized fluorophores. For techniques requiring a pulsed laser source, a supercontinuum fiber laser (SuperK Extreme EXB-4, with multi-line tunable filter SuperK SELECT UV/VIS-nIR, both NKT Photonics) is also implemented. Excitation intensities are mainly controlled by an acousto-optical tuneable filter (AOTF, Polychromatic Modulator 450 - 700 nm, AOTFnC-VIS-TN, with 4-channel RF driver AA.MPDS4C, both AA OPTO-ELECTRONIC) allowing for the free selection of up to eight laser lines from 400 to 675 nm, which can be used separately as well as in any combination. The CW lasers pass respective clean-up filters (Laser Clean-Up Filter ZET 405/20, F49-405; Laser Clean-up Filter ZET 488/10, F49-488; 560/14 BrightLine HC, F39-561; Laser Clean-up Filter ZET 640/10, F49-643; all AHF analysentechnik AG) and are combined into one beam path using beam splitters (BS1: Beamsplitter HC BS 555, F38-555; BS2: Laser Beamsplitter zt 488 RDC, F43-088; BS3: Laser Beamsplitter H 405 LPXR, F48-403; all AHF analysentechnik AG). Magnetic mirror mounts (M1-3: MDI-HS-3030-M6 on RD-MP magnetic plates, all Radiant Dyes) are used throughout the setup to change excitation or emission paths.

1.2 Coupling module

After passing optional neutral density filters, all lasers are coupled into a single mode fiber (Polarization-Maintaining FC/PC Fiber Optic Patch Cable PM-S405-XP, P1-405BPM-FC-2, Thorlabs) with aspheric lens L1 (350 - 700 nm, f = 11.0 mm, NA = 0.25 Aspheric Lens, C220TMD-A, Thorlabs) for uniform, spatially cleaned-up Gaussian laser profiles. Fiber de-coupling is done with achromatic lens L2 (Ø1" Achromatic Doublet, ARC: 400-700 nm, f=30 mm, AC254-030-A-ML, Thorlabs) collimating the excitation beam to a diameter of 10 mm. Afterwards, additional optical elements like linear polarizers (Mounted GT Polarizer, 10 mm x 10 mm, 350 - 700 nm AR Coating, GTH10M-A, Thorlabs) and/or retardation plates (Ø1/2" Mounted Achromatic Half-Wave Plate, Ø1" Mount, 400 - 800 nm, AHWP05M-600, Thorlabs) can be introduced to control the polarization for respective experiments (e.g. anisotropy measurements). An xyz-adjustable lens L3 (Ø1" UVFS Plano-Convex Lens, f = 150.0 mm, ARC: 350 - 700 nm, LA4874-A-ML, Thorlabs) can be pushed into the beam path, which focuses on the back focal plane of the microscope objective, thus allowing switching between either confocal, or epi-fluorescence/TIRFM/VAEM illumination. For VAEM, the angle can be adjusted by lateral translation of this lens. Laser excitation is reflected by the multi-band

beam splitter BS4 (TIRF Quad zt405/488/561/640rpc, F73-410, AHF analysentechnik AG), and directed upwards by a 45° mirror.

1.3 Sample stage

The sample stage mainly consists of a custom-manufactured aluminum frame that holds a piezo table (3-Axis Piezo Scanner with Direct Position Measuring, P-527.3CD, controlled by 3-Axis Digital Piezo Nanopositioning Controller, E-710.3CD, both Physik Instrumente (PI)) for sample-scanning in confocal measurements or small adjustments of the sample position in widefield applications. Laser excitation passes the microscope objective (Objective alpha Plan-Fluar 100x/1,49 Oil M27, 421190-9800-000, Carl Zeiss Microscopy) that can be adjusted in z for focusing onto the sample. An aluminum plate is mounted onto the piezo table with a round recess for the objective, allowing to install different kinds of sample holders. Fluorescence from the sample is collected by the same objective and passes the beam splitter BS4, entering the emission path.

1.4 Detection module

Here, light from the main emission beam path can be redirected via removable mirrors M2 and M3 to the individual detectors. For widefield applications, M2 directs the emission to an sCMOS camera (Digital CMOS camera, ORCA-Flash4.0 V2, Hamamatsu Photonics), focused by an achromatic lens (Ø1" Achromatic Doublet, ARC: 400-700 nm, f=100.0 mm, AC254-100-A-ML, Thorlabs). If needed, green/red channel splitting can be done by the alternative setup shown in Supplemental Figure 1, using beam splitters (BS5: Laser Beamsplitter H 560 LPXR superflat, F48-559; BS6: Shortpass beamsplitter HC BS 556 SP, F38-556; both AHF analysentechnik AG) and two lenses L4 (Biconvex lens; N-BK 7; D=25.4; F=100; mounted, G063854000, Qioptiq) to focus the two emission beams on laterally displaced regions on the camera chip.
For spectroscopy using confocal illumination, the next detector is a spectrograph, consisting of a 30 cm focal length imaging monochromator (Imaging Spectrograph, Acton SP-2356, Princeton Instruments) and a thermoelectrical cooled CCD camera (Digital CCD Camera System, PIXIS 100B, Princeton Instruments), with achromatic lens L5 (Achr. VIS ARB2; D=12.5; F=40; mounted, G052010000, Qioptiq) focusing on the entrance slit.

The following detector block features single-photon avalanche diodes (SPADs, PDM, Micro Photon Devices) for their use in confocal techniques like fluorescence lifetime imaging microscopy (FLIM), fluorescence (lifetime) correlation spectroscopy (F(L)CS), antibunching or other experiments with single-molecule detection. Both SPAD and pulsed laser synchronization signals are fed into a time-correlated single photon counting (TCSPC) module (PicoHarp 300, Picoquant GmbH) to measure the time delay between sample excitation and the arrival of an emitted photon at the detector. Insets a-e in Supplemental Figure 1 describe different configurations using different lenses (L6: Ø1" Achromatic Doublet, ARC: 400-700 nm, f=40.0 mm, AC254-040-A, Thorlabs; L7: Achr. VIS ARB2; D=25.4; F=200; mounted, G063237000, Qioptiq; L8: Achr. VIS ARB2; D=25.4; F=400, G322340322, Qioptiq) for specific experimental setups: (a) dual-color FLIM/F(L)CS with beam splitter BS7 (Laser Beamsplitter H 560 LPXR superflat, F48-559, AHF analysentechnik AG); (b) anisotropy measurements with polarizing beam splitter BS8 (20 mm Polarizing Beamsplitter Cube, 420 - 680 nm, PBS201, Thorlabs); (c) low intensity measurements (no optical sectioning to maximize signal intensity); (d) high-contrast imaging (pinhole for optical sectioning); (e) compromise between (c) and (d), the projected Airy disk is about the size of the SPAD’s detector area, effectively excluding most out-of-focus light.

1.5 Hardware control

All lasers are controlled (directly and via the AOTF) by a custom-written software in LabVIEW (LabVIEW 2018, National Instruments). A standard PC gaming controller is used for focusing and (for widefield applications) lateral adjustments of the sample position, also written in LabVIEW. Widefield sCMOS camera data is recorded with the HoKaWo software (HoKaWo 3.0, Hamamatsu Photonics), while TCSPC data is acquired with SymPhoTime 64 (SPT64-1+2, Picoquant GmbH).

References

zur Oven-Krockhaus, S. (2021). A modular, comprehensive microscopy platform for modern live cell imaging. Thesis. https://doi.org/10.15496/publikation-62250.
