# Supplementary material for: OneFlowTraX: a user-friendly software for super-resolution analysis of single-molecule dynamics and nanoscale organization
Source: Front Plant Sci. 2024 Apr 19;15:1358935. doi: 10.3389/fpls.2024.1358935 (PMC11066300; doi:10.3389/fpls.2024.1358935)
Supplement: Supplementary file 3 [file Table_1.docx]

Supplementary Table 1. Photophysical characteristics of the fluorophores mEos2, mEos3.2, PA‑GFP and PATagRFP.
For the mEos fluorophores, both the native green (G) and the photoconverted red (R) form are listed, while for PA‑GFP and PATagRFP, only the activated forms are presented. The molar attenuation coefficient (ε) refers to the excitation maximum, brightness is the product QY∙ε (for comparison: EGFP features a brightness of 33.6 mM^-1^ cm^-1^). Mean label density was compared in HeLa cells. Note that according to the referenced publication, the high label density of mEos3.2 (compared with mEos2) is likely due to its better maturation and folding properties.

|  | mEos2 (G) | mEos2 (R) | mEos3.2 (G) | mEos3.2 (R) | PA‑GFP | PATagRFP |
| --- | --- | --- | --- | --- | --- | --- |
| Excitation maximum (nm) | 506 | 573 | 507 | 572 | 504 | 562 |
| Emission maximum (nm) | 519 | 584 | 516 | 580 | 517 | 595 |
| Quantum yield | 0.84 | 0.47 | 0.84 | 0.55 | 0.79 | 0.38 |
| ε (M^-1^ cm^-1^) | 56 000 | 41 300 | 63 400 | 32 200 | 17 400 | 66 000 |
| Brightness (mM^-1^ cm^-1^) | 47 | 19 | 53 | 18 | 14 | 25 |
| pH stability (pK_a_) | 5.6 | 6.0 | 5.4 | 5.8 | n.d. | 5.3 |
| Photostability t_½_ (s) | 14.0 | 48.0 | 12.6 | 48.0 | n.d. | 180 |
| Oligomerisation K_d_ (µM) | 20 | 20 | - | - | - | - |
| Mean label density (µm^-2^) | n.d. | 2 649 | n.d. | 9 865 | n.d. | n.d. |
| Reference for measured values | (Zhang et al., 2012) | (Zhang et al., 2012) | (Zhang et al., 2012) | (Zhang et al., 2012) | (Patterson and Lippincott-Schwartz, 2002) | (Subach et al., 2010) |

References

**Patterson, G.H., and Lippincott-Schwartz, J.** (2002). A photoactivatable GFP for selective photolabeling of proteins and cells. Science **297**:1873-1877. <https://doi.org/10.1126/science.1074952>.

**Subach, F.V., Patterson, G.H., Renz, M., Lippincott-Schwartz, J., and Verkhusha, V.V.** (2010). Bright Monomeric Photoactivatable Red Fluorescent Protein for Two-Color Super-Resolution sptPALM of Live Cells. J. Am. Chem. Soc. **132**:6481-6491. <https://doi.org/10.1021/ja100906g>.

**Zhang, M.S., Chang, H., Zhang, Y.D., Yu, J.W., Wu, L.J., Ji, W., Chen, J.J., Liu, B., Lu, J.Z., Liu, Y.F., et al.** (2012). Rational design of true monomeric and bright photoactivatable fluorescent proteins. Nat. Methods **9**:727-729. <https://doi.org/10.1038/Nmeth.2021>.
