# Supplementary material for: OneFlowTraX: a user-friendly software for super-resolution analysis of single-molecule dynamics and nanoscale organization
Source: Front Plant Sci. 2024 Apr 19;15:1358935. doi: 10.3389/fpls.2024.1358935 (PMC11066300; doi:10.3389/fpls.2024.1358935)
Supplement: Supplementary file 4 [file Table_2.docx]

Supplementary Table 2. Nucleotide sequences of the codon-optimized fluorophores mEos3.2, PA‑GFP and PATagRFP.
Shown are the codon optimized nucleotide sequences of the fluorophores mEos3.2, PA-GFP and PATagRFP and the respective position. Genes were synthesized by Invitrogen’s GeneArt services (Thermo Fisher Scientific)

| **mEos3.2**  1 ATG TCT GCT ATC AAG CCT GAT ATG AAG ATC AAG CTC AGG ATG GAA GGT AAC 52 GTG AAC GGA CAC CAC TTC GTG ATC GAT GGT GAT GGA ACT GGA AAG CCT TTC 103 GAG GGA AAG CAG TCT ATG GAT CTT GAG GTG AAA GAG GGT GGA CCT CTC CCT 154 TTC GCT TTC GAT ATT CTC ACT ACC GCT TTC CAC TAC GGA AAC AGG GTG TTC 205 GCT AAG TAC CCT GAT AAC ATC CAG GAT TAC TTC AAG CAG TCT TTC CCA AAG 256 GGA TAC TCT TGG GAG AGA TCA CTC ACC TTC GAG GAT GGT GGA ATC TGC AAC 307 GCT AGG AAC GAT ATT ACT ATG GAA GGT GAT ACC TTC TAC AAC AAG GTG AGG 358 TTC TAC GGA ACT AAC TTC CCT GCT AAC GGA CCT GTG ATG CAG AAA AAG ACT 409 CTC AAG TGG GAG CCT TCT ACC GAG AAG ATG TAC GTG AGA GAT GGT GTG CTC 460 ACT GGT GAT ATT GAG ATG GCA CTT CTC TTG GAG GGA AAC GCT CAC TAC AGA 511 TGC GAT TTC AGG ACC ACC TAC AAG GCT AAA GAA AAG GGT GTG AAG CTC CCT 562 GGT GCT CAC TTC GTT GAT CAT TGC ATC GAG ATC CTC TCA CAC GAT AAG GAT 613 TAC AAC AAA GTT AAG CTC TAC GAG CAC GCT GTG GCT CAT TCT GGA CTT CCA 664 GAT AAC GCT AGA AGA TAA |
| --- |
| **PA-GFP**  1 ATG GTG AGC AAG GGC GAA GAG TTG TTC ACT GGT GTT GTT CCT ATC CTC GTT 52 GAG CTT GAC GGT GAT GTG AAC GGG CAT AAG TTC TCC GTT TCT GGT GAA GGT 103 GAG GGT GAT GCT ACT TAC GGA AAG CTC ACC CTC AAG TTC ATC TGT ACC ACT 154 GGA AAG CTC CCT GTG CCT TGG CCT ACT CTC GTT ACC ACT TTC TCT TAC GGG 205 GTG CAA TGC TTC AGC AGA TAC CCT GAT CAT ATG AAG CAG CAC GAC TTC TTC 256 AAG AGC GCT ATG CCT GAG GGA TAC GTG CAA GAG AGA ACC ATT TTC TTC AAG 307 GAC GAC GGG AAC TAC AAG ACC AGA GCT GAG GTT AAG TTC GAA GGT GAC ACC 358 CTC GTG AAC AGG ATC GAG CTT AAG GGC ATC GAC TTC AAA GAG GAC GGA AAC 409 ATC CTC GGG CAC AAG TTG GAG TAC AAC TAC AAC AGC CAC AAC GTG TAC ATC 460 ATG GCC GAC AAG CAG AAG AAC GGC ATC AAG GCC AAC TTC AAG ATC AGG CAC 511 AAC ATC GAG GAT GGC TCT GTT CAG CTC GCT GAT CAT TAC CAG CAG AAC ACC 562 CCT ATT GGA GAT GGA CCT GTT CTT CTC CCT GAC AAC CAC TAC CTT AGC CAC 613 CAG AGC AAG TTG AGC AAG GAC CCT AAT GAG AAG AGG GAC CAC ATG GTG CTC 664 TTG GAG TTT GTT ACT GCT GCT GGA ATC ACC CTC GGA ATG GAC GAG CTT TAC 715 AAG TGA |
| **PATagRFP**  1 ATG GAA CTC ATC AAA GAA AAC ATG CAC ATG AAG CTC TAC ATG GAA GGG ACC 52 GTG AAC AAC CAC CAT TTC AAG TGC ACA AGC GAA GGT GAG GGA AAG CCT TAC 103 GAG GGA ACT CAG ACC ATG AGA ATC AAG GTG GTG GAA GGT GGA CCT CTT CCT 154 TTC GCC TTC GAT ATT CTC GCC ACC TCC TTC ATG TAC GGG TCC TCT ACT TTC 205 ATC AAC CAC ACT CAG GGA ATC CCG GAC TTC TGG AAG CAA TCT TTT CCA GAG 256 GGA TTC ACC TGG GAG AGA GTG ACT ACT TAC GAG GAT GGT GGT GTG CTC ACT 307 GCT ACT CAG GAT ACT TCT CTT CAG GAC GGC TGC CTC ATC TAC AAC GTG AAG 358 ATC AGA GGT GTG AAC TTC CCG TCT AAC GGA CCG GTG ATG AAG AAA AAG ACT 409 CTC GGA TGG GAG CCG TCT ACC GAG AAA CTT AAG CCT GCT GAT GGT GGA CTT 460 GAG GGA AGA GTT GAC ATG GCT CTT AAG CTC GTT GGA GGT GGA CAT CTC ATC 511 TGC AAC TTC AAG ACC ACC TAC AGG TCT AAG AAG CCG GCC AAG AAC CTT AAG 562 ATG CCT GGG GTT TAC TAC GTG GAC AGG CGT CTT GAG ATT ATC AAA GAG GCC 613 GAC AAA GAG ACT TAC TGG GAG CAG CAT GAA GTG GCT GTG GCT AGG TAT TCT 664 GAC CTT CCA TCT AAG CTC GGG CAC AAG CTC AAT TGA |
