# Supplementary material for: OneFlowTraX: a user-friendly software for super-resolution analysis of single-molecule dynamics and nanoscale organization
Source: Front Plant Sci. 2024 Apr 19;15:1358935. doi: 10.3389/fpls.2024.1358935 (PMC11066300; doi:10.3389/fpls.2024.1358935)
Supplement: Supplementary file 5 [file Table_3.docx]

Supplementary Table 3. Statistical information about applied tests for the respective figures.
All protein fusions or treatment conditions are listed with the number of replicates (n) and the median diffusion coefficient (in µm²/s). In addition, all compared combinations and the corresponding p-value based on the applied test are shown.

| **Figure 5 (*N. benthamiana*)** | | | | | |
| --- | --- | --- | --- | --- | --- |
| Protein fusion | | n | | Median diffusion coefficient [µm²/s] | |
| BRI1-mEos3.2 | | 21 | | 0.0033 | |
| BRI1-PA-GFP | | 21 | | 0.0053 | |
| BRI1-PATagRFP | | 12 | | 0.0040 | |
| Comparisons Figure 5 (*N. benthamiana*) | | | | | |
| Protein 1 | Protein 2 | | p-Value | | Applied test |
| BRI1-mEos3.2 | BRI1-PA-GFP | | 0.0002 | | Kruskal-Wallis, Steel-Dwass |
| BRI1-mEos3.2 | BRI1-PATagRFP | | 0.0797 | |  |
| BRI1-PA-GFP | BRI1-PATagRFP | | 0.0505 | |  |
|  | | | | | |
| **Figure 5 (*A. thaliana*)** | | | | | |
| Protein fusion | | n | | Median diffusion coefficient [µm²/s] | |
| RLP44-mEos3.2 | | 35 | | 0.0106 | |
| RLP44-PA-GFP | | 34 | | 0.0105 | |
| RLP44-PATagRFP | | 34 | | 0.0092 | |
| *Comparisons Figure 5 (A. thaliana)* | | | | | |
| Protein 1 | Protein 2 | | p-Value | | Applied test |
| RLP44-mEos3.2 | RLP44-PA-GFP | | 0.9703 | | Kruskal-Wallis, Steel-Dwass |
| RLP44-mEos3.2 | RLP44-PATagRFP | | 0.0181 | |  |
| RLP44-PA-GFP | RLP44-PATagRFP | | 0.0180 | |  |
|  | | | | | |
| **Figure 6A** | | | | | |
| Set | | n | | Median diffusion coefficient [µm²/s] | |
| LTi6a-mEos2 - Root | | 70 | | 0.0139 | |
| PIP2;1-mEos2 - Root | | 37 | | 0.0032 | |
| LTi6a-mEos2 - Hypocotyl | | 68 | | 0.0540 | |
| PIP2;1-mEos2 - Hypocotyl | | 63 | | 0.0042 | |
| *Comparisons Figure 6A* | | | | | |
| Set 1 | Set 2 | | p-Value | | Applied test |
| LTi6a-mEos2 - Root | PIP2;1-mEos2 - Root | | ≤ 0.0001 | | Kruskal-Wallis, Steel-Dwass |
| LTi6a-mEos2 - Root | LTi6a-mEos2 - Hypocotyl | | ≤ 0.0001 | |  |
| LTi6a-mEos2 - Root | PIP2;1-mEos2 - Hypocotyl | | ≤ 0.0001 | |  |
| PIP2;1-mEos2 - Root | LTi6a-mEos2 – Hypocotyl | | ≤ 0.0001 | |  |
| PIP2;1-mEos2 - Root | PIP2;1-mEos2 - Hypocotyl | | ≤ 0.0001 | |  |
| PIP2;1-mEos2 - Hypocotyl | LTi6a-mEos2 - Hypocotyl | | ≤ 0.0001 | |  |
|  | | | | | |
| **Figure 6B** | | | | | |
| Set | | n | | Median diffusion coefficient [µm²/s] | |
| Light | | 14 | | 0.0117 | |
| Dark, upper | | 26 | | 0,0177 | |
| Dark, lower | | 27 | | 0,0103 | |
| *Comparisons Figure 6B* | | | | | |
| Set 1 | Set 2 | | p-Value | | Applied test |
| Light | Dark, upper | | 0.0094 | | Kruskal-Wallis, Steel-Dwass |
| Light | Dark, lower | | 0.0604 | |  |
| Dark, upper | Dark, lower | | ≤ 0.0001 | |  |
|  | | | | | |
| **Figure 6C** | | | | | |
| Set | | n | | Median diffusion coefficient [µm²/s] | |
| Light, 0 mM | | 14 | | 0.0117 | |
| Light, 100 mM | | 18 | | 0.0139 | |
| Light, 300 mM | | 22 | | 0.0209 | |
| Dark, 0 mM | | 16 | | 0.0199 | |
| Dark, 100 mM | | 13 | | 0.0215 | |
| Dark, 300 mM | | 16 | | 0.0271 | |
| *Comparisons Figure 6C (light and dark evaluated separately)* | | | | | |
| Set 1 | Set 2 | | p-Value | | Applied test |
| Light, 0 mM | Light, 100 mM | | 0.3683 | | Kruskal-Wallis, Steel-Dwass |
| Light, 0 mM | Light, 300 mM | | ≤ 0.0001 | |  |
| Light, 100 mM | Light, 300 mM | | 0.0058 | |  |
| Dark, 0 mM | Dark, 100 mM | | 0.6686 | |  |
| Dark, 0 mM | Dark, 300 mM | | 0.0011 | |  |
| Dark, 100 mM | Dark, 300 mM | | 0.0687 | |  |
