# Supplementary material for: OneFlowTraX: a user-friendly software for super-resolution analysis of single-molecule dynamics and nanoscale organization
Source: Front Plant Sci. 2024 Apr 19;15:1358935. doi: 10.3389/fpls.2024.1358935 (PMC11066300; doi:10.3389/fpls.2024.1358935)
Supplement: Supplementary file 6 [file Table_4.docx]

Supplementary Table 4. List of sptPALM acquisition and analysis parameters.

|  |  | *N. benthamiana* constructs with | | |  | *A. thaliana* constructs with | | |
| --- | --- | --- | --- | --- | --- | --- | --- | --- |
|  |  | mEos3.2 | PA‑GFP | PATagRFP |  | mEos3.2 | PA‑GFP | PATagRFP |
| acquisition |  |  |  |  |  |  |  |  |
| Power 405 nm (µW) |  | <15 | <15 | <15 |  | <20 | <20 | <40 |
| Power 488 nm (µW) |  |  | 600 |  |  |  | 900 |  |
| Power 561 nm (µW) |  | 1800 |  | 1300 |  | 1800 |  | 2000 |
| frame rate (Hz) |  | 20 | 20 | 20 |  | 50 | 20 | 20 |
| number of frames |  | 2500- 5000 | 2500- 5000 | 2500- 5000 |  | 5000 | 2500 | 2500 |
|  |  |  |  |  |  |  |  |  |
| localization |  |  |  |  |  |  |  |  |
| conversion |  | 0.48 | 0.48 | 0.48 |  | 0.48 | 0.48 | 0.48 |
| offset |  | 100 | 100 | 100 |  | 100 | 100 | 100 |
| pixel size |  | 100 | 100 | 100 |  | 100 | 100 | 100 |
| filter size |  | 1.2 | 1.2 | 1.2 |  | 1.2 | 1.3 | 1.3 |
| cut-off |  | 3 | 3 | 3 |  | 3 | 3.5 | 4 |
| PSF ROI size |  | 7 | 7 | 7 |  | 7 | 7 | 7 |
|  |  |  |  |  |  |  |  |  |
| tracking |  |  |  |  |  |  |  |  |
| max. linking distance |  | 200 | 200 | 200 |  | 200 | 200 | 200 |
| max. gap frames |  | 4 | 4 | 4 |  | 4 | 4 | 4 |
|  |  |  |  |  |  |  |  |  |
| mobility analysis |  |  |  |  |  |  |  |  |
| min. number of datapoints |  | 8 | 8 | 8 |  | 8 | 8 | 8 |
| number of points for linear fit |  | 4 | 4 | 4 |  | 4 | 4 | 4 |
| adj. R² fit threshold value |  | 0.4 | 0.4 | 0.4 |  | 0.4 | 0.4 | 0.4 |
|  |  |  |  |  |  |  |  |  |
