# Supplementary material for: OneFlowTraX: a user-friendly software for super-resolution analysis of single-molecule dynamics and nanoscale organization
Source: Front Plant Sci. 2024 Apr 19;15:1358935. doi: 10.3389/fpls.2024.1358935 (PMC11066300; doi:10.3389/fpls.2024.1358935)
Supplement: Supplementary file 7 [file Table_5.docx]

Supplementary Table 5. List of constructs.
All used constructs are listed with our internal numbering, their name, their purpose and source.

| Internal # | Name | Purpose | Source |
| --- | --- | --- | --- |
| B1-E1 | pJET1.2_pBRI1 | LI A-B Module | this study |
| 4390 | pJET1.2_pRLP44 | LI A-B Module | this study |
| B1-E3 | pJET1.2_cBRI1 | LI B-D Module | this study |
| 4401 | pJET1.2_cRLP44 | LI B-D Module | this study |
| 4392 | pJET1.2_(GS)mEos3.2 | LI D-E Module (with Gly/Ser Linker) | this study |
| 4441 | pJET1.2_(GA)mEos3.2 | LI D-E Module (with Gly/Ala Linker) | this study |
| B1-D2 | pJET1.2_(GS)PA-GFP | LI D-E Module (with Gly/Ser Linker) | this study |
| B1-C8 | pJET1.2_(GS)PATagRFP | LI D-E Module (with Gly/Ser Linker) | this study |
| 4437 | pJET1.2_(GA)PA-GFP | LI D-E Module (with Gly/Ala Linker) | this study |
| 4438 | pJET1.2_(GA)PATagRFP | LI D-E Module (with Gly/Ala Linker) | this study |
| 4398 | pUC57_nosTerm | LI E-F Module | (Binder et al., 2014) |
| 4400 | pGEM-T_pFAST | LI F-G Module | this study |
| 4503 | pUC57_Hygromycin | LI F-G Module | (Binder *et al.*, 2014) |
|  |  |  |  |
| B1-E8 | BB10 -- pBRI1:cBRI1-(GS)mEOS3.2-nosTerm-pFast -- BB10 | LII; Expression in *N. benthamiana* | this study |
| B1-F8 | BB10 -- pBRI1:cBRI1-(GS)PA-GFP-nosTerm-pFast -- BB10 | LII; Expression in *N. benthamiana* | this study |
| B1-E9 | BB10 -- pBRI1:cBRI1-(GS)PATagRFP-nosTerm-pFast -- BB10 | LII; Expression in *N. benthamiana* | this study |
| 4418 | BB10 -- pRLP44:cRLP44-(GS)mEOS3.2-nosTerm-pFast -- BB10 | LII; Expression in *A. thaliana*; for Figure 6 | this study |
| 4446 | BB10 -- pRLP44:cRLP44-(GA)mEOS3.2-nosTerm-pFast -- BB10 | LII; Expression in *A. thaliana*; for Figure 5 | this study |
| 4479 | BB10 -- pRLP44:cRLP44-(GA)PA-GFP-nosTerm-pFast -- BB10 | LII; Expression in *A. thaliana*; for Figure 5 | this study |
| 4494 | BB10 -- pRLP44:cRLP44-(GA)PATagRFP-nosTerm-Hygromycin -- BB10 | LII; Expression in *A. thaliana*; for Figure 5 | this study |

References

**Binder, A., Lambert, J., Morbitzer, R., Popp, C., Ott, T., Lahaye, T., and Parniske, M.** (2014). A modular plasmid assembly kit for multigene expression, gene silencing and silencing rescue in plants. PLoS One **9**:e88218. <https://doi.org/10.1371/journal.pone.0088218>.
